# The effects of COVID-19 on training within urology: Lessons learned in virtual learning, human factors, non-technical skills and reflective practice

**DOI:** 10.1177/2051415820950109

**Published:** 2021-01

**Authors:** T Fonseka, R Ellis, H Salem, PA Brennan, T Terry

**Affiliations:** 1 Urology Department, Royal Derby Hospital, University Hospitals of Derby and Burton, UK; 2 Maxillofacial Unit, Queen Alexandra Hospital, UK; 3 Urology Department, Nottingham University Hospitals, UK

**Keywords:** Human factors, training, education, simulation, safety, reflective practice

## Abstract

The COVID-19 pandemic has changed training and recruitment in urology in unprecedented ways. As efforts are made to ensure trainees can continue to progress, lessons can be learned to improve training and urological practice even after the acute phase of the pandemic is over. Novel methods of education through virtual learning have burgeoned amidst the social distancing the pandemic has brought. The importance of training in human factors and non-technical skills has also been brought to the fore while operating under the constraints of personal protective equipment and working in new teams and unfamiliar environments. This paper critically appraises the available evidence of how urological training has been affected by COVID-19 and the lessons we have learned and continue to learn going forward.

**Level of Evidence:**

Not Applicable.

## Introduction

COVID-19 has had a profound effect globally as well as on UK health services, with urology being no exception. Service delivery has required extensive re-configuration with large-scale cancellation of elective operating and a vast reduction in face-to-face clinics. The pandemic has caused uncertainty amongst trainees, with considerable changes being made to training and national recruitment as well as the Membership (MRCS) and Fellowship (FRCS) of the Royal College of Surgeons examinations required to progress in surgery and to gain a certificate of completion of training (CCT). However, amongst the challenges this pandemic has caused, lessons have been and continue to be learned for the benefit of urological training. Certainly, virtual learning has come to the fore in ways never seen previously.

Furthermore, operating in the challenging environment of COVID-19 has also highlighted the impact of human factors (HF) and non-technical skills training, especially in reducing medical error.

In this paper we critically appraise the current available evidence on changes to training and recruitment within urology during the pandemic and the effects this has had on the workforce. We discuss the value of virtual learning, the importance of HF, non-technical skills training and reflective practice.

## Effects on training

### Urology trainees

For those already in urology training the abrupt reduction in elective operating and outpatient clinics has drastically limited training opportunities within the specialty. Amparore et al.^1^ have described Italian urology trainees experiencing similar on-call responsibilities but noted a significant reduction in exposure to outpatient visits, diagnostic procedures and surgery including endoscopic, open and minimally invasive procedures. During the pandemic the proportion of trainees who experienced severe reduction (>40%) or complete suppression (>80%) of exposure ranged from 41.1% to 81.2% for clinical activity and from 44.2% to 62.1% for surgical activity. At our institution the experience has been similar, with the vast majority of outpatient appointments converted to telephone clinics, mostly conducted by consultants. All elective operations were initially postponed unless considered high priority, such as obstructing stone disease or certain high-grade cancers. Much of the emergency operating has also been conducted by consultants rather than registrars so as to minimise time in theatre, as per guidance issued by the Royal College of Surgeons (RCS). This has understandably caused considerable impairment to training. Given the recent curriculum changes towards a more competency-based system, it is conceivable that the reduction in operating opportunities may result in training extensions for some trainees. However, one advantage of this new curriculum is that if a trainee has already met the required competencies, they are unlikely to be delayed in achieving their CCT despite the reduction in elective work.

With the large-scale changes to operative and clinical practice, one may argue that managing patients within the COVID-19 pandemic can also present new and unforeseen training opportunities; for example, the ability to learn skills in crisis management, healthcare management and leadership skills. This has potential to result in more well-rounded trainees who have developed in a range of different aspects of practice. Yet, there is no denying that many trainees would have experienced difficulty in achieving expected competencies at Annual Review of Competency Progression (ARCP). Accordingly, the Joint Committee on Surgical Training (JCST) have responded by creating a ‘no fault’ outcome to ARCP, known as ‘Outcome 10’,^2^ whereby trainees who have been unable to meet training level requirements can have their training extended or be permitted to progress despite not all competencies being achieved in time. For example, for core trainees under normal circumstances achieving MRCS is a pre-requisite to gaining a place on ST3 training. Yet due to COVID-19, the MRCS part B exam has not been conducted and therefore core trainees who have gained a place on ST3 training but have been unable to achieve MRCS can still be allowed to continue to ST3 and sit the MRCS part B exam when it is next available. In this way ARCP outcomes have been adapted to account for the changes in competencies.

### Junior doctors

For junior doctors applying to UK urology training through national selection, the face-to-face interview component of the recruitment process has been removed completely, with applicants scored solely on online self-assessment.^3^ Applicants go through a series of questions online pertaining to different areas of practice including quality improvement projects, presentations, publications, courses, teaching experience, surgical logbook, prizes and leadership roles, and score themselves in each category. Therefore, as there is no face-to-face interview, be it in person or virtually, there is no formal way of assessing key components such as communication, technical operative skills, and portfolio review. There is little evidence of the validity of using solely self-assessment as a selection method and the limitations of self-assessment have been well documented.^4^ Only time will tell the impact of removing the interview process from national selection. Recent studies have validated the use of alternative selection methods including MRCS pass scores, which may reduce the need for face-to-face aspects of selection in future years.^5^

Core surgical trainees will undoubtedly experience difficulties in gaining exposure to emergency and elective urology over the next few months due to reduced clinical activity and the redeployment of many to staff wards occupied mostly by patients with COVID-19. With such disruption to core training programmes this gap in experience and opportunity may need to be taken into account in future ST3 national selection processes in order to ensure a fair and non-discriminatory process. Foundation doctors may find themselves in a similar position when applying for run-through ST1 posts next year with little experience or exposure to urology.

### Medical students

At medical student level, almost all clinical attachments have been cancelled, which will likely have profound effects on recruitment to urology in the future.^6^ Urology is a unique specialty that is often under-represented in medical school curricula; in the USA only 5% of medical schools have a mandatory urology rotation. It is possible that without urology exposure, students will be less inclined to choose it as their preferred specialty, which could further adversely affect competition rates at national selection, which have been steadily declining in recent years.^7^ Before the pandemic there was arguably already a relative lack of undergraduate training in urology,^8,9^ with many junior doctors feeling a lack of confidence in performing basic urological clinical skills such as catheterisation.^10-12^

The current reduction in exposure to urology at medical student level may exacerbate this lack in training. Delaying recruitment and allowing students time to complete urology placements may be the best way forward. Remote training opportunities provided by Royal Colleges and Medical Associations should be taken advantage of by students and prospective surgeons. These opportunities may have unforeseen benefits, such as reducing the exorbitant costs of mandatory face-to-face training courses, potentially widening access to surgical training programmes.^13^

### Virtual learning

Telemedicine and the use of technology can be of great benefit in maintaining training during a pandemic. In the USA, Vargo et al.^14^ have created a structured framework through the use of virtual learning to continue urology training despite lower surgical volumes and the inability to have face-to-face meetings during the pandemic. The team used online platforms to provide an educational activity for each week day. These activities included discussing American Urological Association updates, holding a virtual journal club, having a guest virtual lecture, discussing interesting cases and going over a chapter of *Campbell’s Urology* textbook.^15^ The authors found this framework worked well to provide an element of normalcy in a time of great uncertainty for trainees while maintaining mental sharpness.

Simulation training can also be beneficial at a time when hands-on training is limited. Urology has been a leading specialty in the use of simulation devices, particularly for education in minimally invasive surgery.^16^ A range of simulation models exist even for use at home, such as with the lower fidelity box trainers.^17^ Though many are still in the experimental stage requiring more robust validity studies to demonstrate transferability of skills, box trainers are relatively simple to create at home and, in conjunction with virtual learning platforms, could be incorporated into a curriculum.^18^ Especially for the more senior trainees who would not want to lose operative skills gained through years of practice, simulation can help maintain these competencies for progression towards CCT.^19^

The JCST have published a statement discussing the importance of continuing professional development throughout the COVID-19 crisis.^20^ Opportunities for training and development are abundant as the medical community continues to learn how to manage patients with COVID-19. Table 1 summarises key online resources related to health and wellbeing, personal protective equipment (PPE) and adapting practice during the COVID-19 pandemic. Royal Colleges are providing free webinars to all healthcare professionals on a weekly basis sharing knowledge, discussing new evidence regarding COVID-19, changes to services, and revision of intensive care principles, physiology and pharmacotherapy.^21-24^ Novel techniques for remote teaching such as videoconferencing are being utilised by such organisations as the BAUS Section of Trainees and the Royal Society of Medicine Urology section to minimise the interruption to our training. These resources have been made free to trainees, and include mock viva examination questions to help with preparation for the Urology FRCS examinations. From this crisis has emerged a new way of training tomorrow’s surgeons and sharing experience that hopefully will continue after the nationwide lockdown has ended. With videoconferencing technology so readily available, we feel that geographical barriers to learning should become a thing of the past.

**Table 1.** Key resources to guide practice in the COVID-19 era.

	Key points	Online resources
**Health and wellbeing**	• Stay connected to friends and family. • Take regular breaks from your phone and computer to avoid information overload. • Do not forget the importance of regular sleep, healthy eating and staying hydrated. • Turn to supervisors and colleagues as it may help to share thoughts and listen to colleagues having similar experiences. • Do not feel pressured to work when unwell. Speak with occupational health for advice.	**RCS** https://www.rcseng.ac.uk/careers-in-surgery/csas/ **BMA** https://www.bma.org.uk/advice-and-support/covid-19/your-health/covid-19-your-wellbeing **GMC** https://www.gmc-uk.org/ethical-guidance/ethical-hub/covid-19-questions-and-answers#Your-health-and-wellbeing
**Personal protective equipment**	• Ensure you have been fit-tested for FFP3 masks stocked in your Trust. • Practice donning and doffing with trainers. • Familiarise yourself with what forms of PPE are to be used in theatre as the guidance may change at rapid pace. • If a range of PPE is offered take time to choose what you feel you can operate in most comfortably and safely. • If PPE is inadequate do not risk compromising patient safety. Inform hospital managers accordingly. • Make use of online non-technical skills and human factors training courses to optimise performance when operating in PPE.	**RCS** https://www.rcsed.ac.uk/professional-support-development-resources/covid-19-resources/protecting-yourself/personal-protective-equipment **BMA** https://www.bma.org.uk/advice-and-support/covid-19/ppe/covid-19-ppe-for-doctors **GMC** https://www.gmc-uk.org/ethical-guidance/ethical-hub/covid-19-questions-and-answers#Working-safely
**Adapting practice to COVID-19**	• Engage with NHS-approved platforms such as Microsoft Teams to aid communication with colleagues in handover and accessing ward lists. • In virtual consultations set an agenda with the patient at the start. Use clear communication and active listening, ensuring that you pick up on cues. • If re-deployed make use of departmental inductions and training as well as online revision tools. • Familiarise yourself with COVID-19-specific consent forms. • Use published guidance to aid triage of non-emergency surgery. • Consult the literature to keep up to date with current practice.	**RCS** https://www.rcseng.ac.uk/standards-and-research/standards-and-guidance/good-practice-guides/coronavirus/ **BMA** https://www.bma.org.uk/advice-and-support/covid-19/adapting-to-covid/covid-19-video-consultations-and-homeworking **GMC** https://www.gmc-uk.org/ethical-guidance/ethical-hub/covid-19-questions-and-answers#Remote-consultations

PPE: Personal protective equipment; RCS: Royal College of Surgeons; BMA: British Medical Association; GMC: General Medical Council.

## Human factors

As a workforce we are becoming more aware of the impact of HF in medical error.^25^ While we cannot compare aviation with medicine, many lessons have been learned from the airline industry and others who have implemented HF awareness and training, following the realisation that 70% of fatal plane crashes were due to human error, not technical faults.

A large recently published systematic review and meta-analysis of 337,000 patients found that one in every 20 hospital admissions resulted in a medical error,^26^ with the majority occurring in either surgery or prescribing.^27,28^ One in 300 of these results in death.^29^ With such high stakes we have a duty to minimise risks as much as possible for our patients. We spend years honing clinical and technical skills in surgery to improve our patient outcomes, but often very little time is spent identifying and addressing the causes of human error. The COVID-19 crisis has seen urologists working in unfamiliar environments and having to make decisions that differ to normal treatment pathways, adding to the already stressful task of providing a safe and effective urology service. Guidance has been issued by the General Medical Council (GMC) with regards to making these decisions, though it is near impossible to alleviate the anxiety that clinicians may experience in working outside of their normal comfort zone.^30^

The GMC’s statement also highlights the need for the profession to look after themselves, enabling clinicians to care for patients.^30^ Many clinicians will be working longer hours and more irregular shift patterns, often in full PPE. As a result, it has never been more important to recognise the need for regular work breaks and recovery days, enabling clinicians to remain well fed, rested and hydrated to reduce the risk of medical errors.^31^ It is important to appreciate the contribution of personal factors to poor decision making in such a stressful environment, which can include being hungry, angry, late or tired (HALT).^32^ Remembering the mnemonic and stopping, even for a short break, when either individuals or teams experience one or more of these personal factors can make such a difference to personal performance and wellbeing.

Tiredness and fatigue play a significant role in poor cognitive functioning and decision making.^33^ This issue was addressed in the new junior doctors’ contract,^34^ with stipulations made regarding the minimum number of hours off duty following on-call shifts. Despite the increase in workload due to COVID-19, NHS Trusts are making every effort to ensure clinicians have adequate time off to prevent tiredness and burnout. Resources have been made available to promote wellbeing for clinicians within Trusts and nationwide as we start to recognise the frequency and impact of burnout in doctors.^35^ Stress and emotional factors often play a role in our decision-making ability, prompting the promotion of resilience training for clinicians.^36,37^

An appreciation for the impact of HF in medical error has been brought into sharp highlight by COVID-19. Working in an unfamiliar and stressful environment undoubtedly increases the risk of medical error. The efforts made nationwide to look after the medical profession have been considerable, and we must aim to continue to focus on the health and wellbeing of colleagues after this crisis abates in order to reduce error and workforce attrition. The RCS is being proactive in this regard.

## Non-technical skills

### Operating

**Figure 1.**
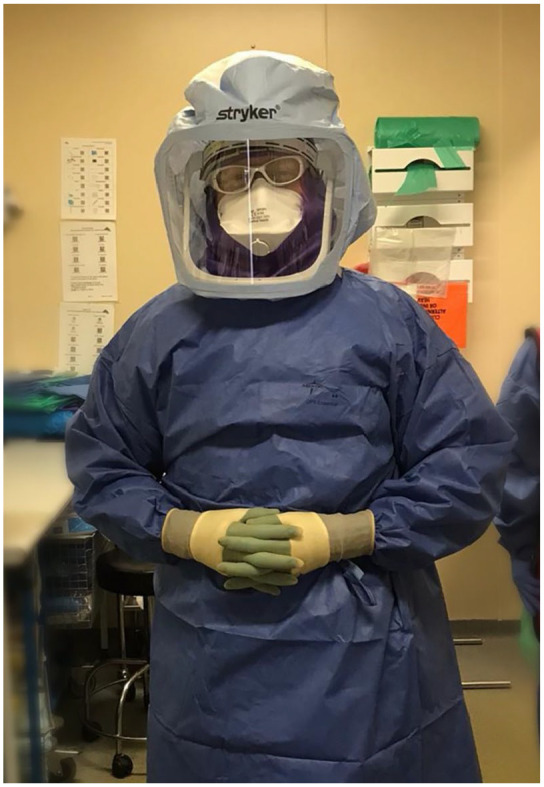
A clinician wearing full personal protective equipment including surgical hood to reduce glare from visor when performing ureteroscopy.

At the time of writing (30 April 2020), all emergency operations are being undertaken with the operating team wearing full PPE. This includes a fitted FFP2/3 facemask, visor, gown and gloves.^38^ Many will have experienced how uncomfortable and unfamiliar this protective equipment is to wear, especially when operating for long periods of time. It significantly reduces one’s situational awareness in theatre by blocking out sounds, reducing hearing as well as peripheral vision. In Figure 1 the clinician pictured opted for a surgical hood instead of more widely available face visors in an effort to reduce glare when performing a ureteroscopy and laser fragmentation of ureteric calculi. The effect full PPE has on both verbal and non-verbal communication between theatre members is profound. An awareness of the impact of these is crucial to the prevention of medical error.^39^ To aid clinicians in the recognition and prevention of such errors the Non-Technical Skills for Surgeons (NOTSS) course^40^ has been made freely available to access online by the Royal College of Surgeons of Edinburgh.^41^ We believe that non-technical skills are crucial in the prevention of surgical error especially while operating at such a challenging time, and that this course should be mandatory for all surgeons, regardless of grade and experience.

### Team working

Working amidst the COVID-19 crisis has seen the flattening of hierarchies within the workforce. This has resulted in a deepened appreciation for colleagues and the expertise that each individual can bring to a team. This crisis has highlighted the ability of the NHS workforce to work as a team united in the pursuit of best patient care and the reduction of medical errors. We have a duty to patients to continue working as a cohesive multi-disciplinary team after the COVID-19 crisis abates, continually striving to improve patient outcomes and reduce medical error.^42,43^

## Reflective practice

Reflective practice (RP) is a critical component for developing expertise in surgeons and trainees, with the aim of promoting excellence in patient care. RP is a conscious effort, using a structured framework, to think about an experience or an event to develop insights, and where necessary affect transitional or transformational changes using a novel paradigm. The simplest RP framework is the Driscoll model,^44^ which is based on three self-aimed questions: ‘What?’, ‘So What?’ and ‘Now What?’. ‘What?’ is a rich description of self-awareness of an experience. ‘So What?’ is an evaluation and analysis of the description of the experience, and ‘Now What?’ is the exit synthesis action plan which may generate a novel way of managing the initial experience with the intention of improving patient care. Such reflective cycles may be iterative as new information becomes available.

At our institution one-third of the urology registrars were re-deployed to work in critical care, and engaging in RP has certainly been found to be beneficial in developing practice going forward. Using the ‘journal entry’ section on the Intercollegiate Surgical Curriculum Programme (ISCP) online portfolio, the registrars who were re-deployed have been able to reflect on the skills and knowledge they have gained through working in a different specialty. RP has enabled them to consolidate learning and how they can apply their experience in critical care to their future practice in urology. This has aided development in training at a time when formal face-to-face training can be scarce, and the authors perceive engaging in RP in this way has helped them use experiences in redeployment to progress towards becoming more well-rounded urologists.

RP also has a major role in making complex decisions in the face of Covid-19 regarding service adaptations, scope of practice, end-of-life care and protecting the workforce. These decisions are considered by multi-professional teams, but RP for individual surgeons and trainees increases self-awareness through being more open minded, being in the moment, using active listening and receiving real-time feedback. These benefits translate into better team working and improvements in patient care during the COVID-19 pandemic. All surgeons should be regular reflective practitioners, either in the moment or on action, and crystallise outcomes in writing (journals, diaries, Twitter, blogs, e-portfolio) or in formal conversation with colleagues, else the new learning may be lost. It is not surprising that RP is a mandatory part of appraisal, revalidation and ARCP.

## Conclusion

The COVID-19 pandemic has seen considerable change in training opportunities in urology with uncertain effects on specialty recruitment. Yet despite the setbacks in training this has caused, we have seen a flourishing of novel concepts in the field of virtual learning in medicine. The importance of HF in surgery and non-technical skills has been brought to the fore in unprecedented ways, and resources have been made widely available to improve in these areas of practice. RP is important for individual surgeons and trainees to manage their new experiences with COVID-19. This global pandemic has undoubtedly changed the way we learn and practise urology, and it is our hope that these lessons will continue to be built upon long after this acute crisis is over.
